# Supplementary material for: Physiological, Productive, and Soil Rhizospheric Microbiota Responses of ‘Santina’ Cherry Trees to Regulated Deficit Irrigation Applied After Harvest
Source: Plants (Basel). 2025 Nov 26;14(23):3611. doi: 10.3390/plants14233611 (PMC12693967; doi:10.3390/plants14233611)
Supplement: Supplementary file 1 [file plants-14-03611-s001.zip › plants-3981439-supplementary.pdf]

Table S1. Monthly mean temperature (°C), relative humidity (%), Vapor Pressure deficit (VPD) (kPa) and month precipitation (mm) Placillas, O'Higgins Region, Chile during the 2021-2022, 2022-2023, and 2023-2024 seasons.

Month	Season			
	Temperature (°C)	Relative humidity (%)	VPD (kPa)	Month precipitation (mm)
Season 2022				
September	10.8	75.5	1.2	39.6
October	13.4	68.0	1.7	5.1
November	16.5	63.5	2.2	0.4
December	18.9	61.6	2.6	2.1
January	19.8	59.2	2.9	0.0
February	19.0	61.1	3.0	0.0
March	15.8	65.3	2.4	0.1
April	11.8	75.6	1.3	52.5
May	8.4	90.5	0.5	33.6
Season 2023				
September	10.1	78.5	1.0	19.7
October	13.5	68.5	1.6	0.3
November	17.5	64.9	2.1	4.1
December	19.8	56.5	3.0	0.6
January	20.3	57.5	3.1	0.0
February	20.2	57.3	3.6	0.0
March	18.3	62.3	3.1	0.0
April	13.7	72.8	1.7	44.4
May	9.8	85.4	0.8	27.6
Season 2024				
September	10.8	80.3	0.8	109.8
October	12.9	70.9	1.3	29.7
November	14.7	71.5	1.5	52.9
December	18.1	62.0	2.4	0.0
January	20.5	63.3	3.0	0.0
February	20.5	64.9	2.9	7.6
March	17.6	65.4	2.6	0.0
April	12.9	75.6	1.4	43.9
May	7.7	83.7	0.7	86.8

The vapor pressure deficit (VPD) was estimated using the monthly mean of daily maximum temperature and the monthly mean of daily minimum relative humidity, simulating the conditions of highest atmospheric demand for each month.
